# Artificial Intelligence in Nuclear Medicine (2015-2025): Clinical Applications, Regulatory Governance, and Pharmacotherapeutic Implications

**DOI:** 10.7759/cureus.113457

**Published:** 2026-07-27

**Authors:** Mariela Alpizar-Rojas, Helena Jaromirski-Segura, Luis Guillermo Herrera-Jiménez, José A Castro-Gamboa, Jeaustin Mora-Jiménez, Sebastián Arguedas-Chacón, Esteban Zavaleta-Monestel

**Affiliations:** 1 Nuclear Medicine, Hospital Clínica Bíblica, San José, CRI; 2 Pharmacy, Universidad de Ciencias Médicas, San José, CRI; 3 Pharmacy, Universidad de Costa Rica, San José, CRI; 4 Pharmacy, Hospital Clínica Bíblica, San José, CRI; 5 Research, Hospital Clínica Bíblica, San José, CRI

**Keywords:** artificial intelligence, dosimetry, medical imaging, nuclear medicine, radiopharmaceuticals, radiopharmacy, theranostics

## Abstract

Artificial intelligence (AI) is becoming increasingly relevant in nuclear medicine, particularly in applications that depend on quantitative image analysis, image reconstruction, acquisition optimization, and clinical decision support. This critical narrative review examines the evolution, clinical applications, ethical and regulatory challenges, and pharmacotherapeutic implications of AI in nuclear medicine from 2015 to 2025. A structured literature search was conducted in PubMed/MEDLINE, Scopus, Web of Science, Google Scholar, and selected professional and regulatory websites, including those of the Society of Nuclear Medicine and Molecular Imaging, the European Association of Nuclear Medicine, the International Atomic Energy Agency, the US Food and Drug Administration, and the European Medicines Agency.

The reviewed literature reports advances in deep learning-based reconstruction, denoising, and preservation of quantitative parameters in positron emission tomography and single-photon emission computed tomography. Clinical applications have expanded across oncology, neurology, cardiology, theranostics, and radiopharmacy-related workflows. However, the evidence remains limited by methodological heterogeneity, predominantly retrospective and single-center designs, restricted external validation, uneven regulatory development, and limited representation of Latin American settings.

Overall, AI may improve diagnostic precision, personalized dosimetry, workflow efficiency, and radiopharmaceutical safety in nuclear medicine. Responsible implementation will require robust multicenter validation, transparent evaluation standards, adaptive governance, and multidisciplinary oversight. Radiopharmacists should be actively involved in this process, particularly in relation to radiopharmaceutical quality, traceability, dosimetry, and pharmacotherapeutic decision-making.

## Introduction and background

Nuclear medicine differs from conventional imaging modalities in its capacity to depict functional, metabolic, and molecular processes rather than anatomical structure alone. Positron emission tomography (PET) and single-photon emission computed tomography (SPECT), increasingly performed within hybrid platforms such as PET/computed tomography (CT), SPECT/CT, and PET/magnetic resonance imaging (MRI), generate quantitative information that is highly sensitive to acquisition protocols, reconstruction parameters, radiopharmaceutical kinetics, and scanner-specific characteristics [1-4]. These features make nuclear medicine central to diagnosis, staging, treatment planning, and response assessment while also creating persistent challenges related to standardization, longitudinal comparability, quantitative reproducibility, and equitable access to advanced molecular imaging.

In this review, quantitative imaging refers to the extraction and interpretation of measurable imaging parameters, such as standardized uptake values, lesion burden, radiomic features, and absorbed-dose estimates. Theranostics refers to the integration of diagnostic imaging and targeted radionuclide therapy, while radiopharmacy refers to the preparation, quality control, traceability, and safe use of radiopharmaceuticals in clinical practice.

Artificial intelligence (AI), particularly machine learning and deep learning, has emerged as a potential approach to address some of these challenges. Neural network architectures, including convolutional neural networks (CNNs), encoder-decoder models, U-Net-derived architectures, and generative adversarial networks (GANs), have been applied to image reconstruction, denoising, lesion segmentation, quantitative harmonization, and dose estimation [1-3,5,6]. In parallel, radiomics has expanded the analytic value of PET and SPECT by enabling the systematic extraction of quantitative image features associated with tumor biology, therapeutic response, and cardiovascular risk [5,6]. Together, these methods extend the interpretive potential of nuclear medicine beyond visual assessment, although their clinical value depends on robust validation and reproducible performance across acquisition settings.

AI applications in nuclear medicine are no longer limited to image processing. Emerging work has explored AI-supported radiopharmacy workflows, including radiochemical synthesis optimization, quality control assistance, process monitoring, and inventory-related planning [7,8]. AI has also been evaluated in theranostic pathways, where quantitative imaging, time-activity modeling, absorbed-dose estimation, and individualized dosimetry are central to radioligand therapy planning [9,10]. These developments have direct implications for radiopharmacists and hospital pharmacists, whose responsibilities increasingly intersect with radiopharmaceutical preparation, verification, traceability, quality assurance, dosimetric interpretation, and pharmacotherapeutic oversight.

Despite this rapid expansion, the evidence base remains methodologically uneven. Many studies are retrospective, single-center, technically focused, or validated under narrow acquisition and reconstruction conditions, limiting their external validity and clinical generalizability [1-3,11-15]. Regulatory frameworks for AI as software as a medical device continue to evolve, and implementation guidance tailored to Latin American health systems remains limited [15-22]. In addition, previous reviews have largely emphasized technical imaging applications, whereas the pharmacotherapeutic, radiopharmacy, and governance dimensions of AI adoption have been less consistently integrated.

This review critically examines the evolution of AI in nuclear medicine from 2015 to 2025. Specifically, it aims to (1) summarize the evolution of AI applications in nuclear medicine imaging; (2) synthesize clinical applications in oncology, neurology, cardiology, theranostics, and radiopharmacy-related workflows; (3) examine ethical, regulatory, and implementation challenges, with attention to Latin American and Costa Rican settings; and (4) discuss pharmacotherapeutic implications and the role of radiopharmacists in patient safety, traceability, dosimetry, and governance.

## Review

Materials and methods

Review Design

This study was designed as a critical narrative review informed by a structured literature search. This framework was selected because AI in nuclear medicine is a heterogeneous and rapidly evolving field, with evidence spanning algorithm development, clinical imaging studies, radiopharmacy applications, professional guidance, and regulatory documents. The aim was not to conduct a systematic review, generate pooled quantitative estimates, or formally grade the quality of evidence, but rather to provide an interpretive synthesis of the literature, identify convergent trends, examine methodological and translational discrepancies, and discuss implications for clinical practice, radiopharmacy, governance, and future research.

A structured search approach was used to improve transparency and breadth in source identification. However, the review was not conducted according to Preferred Reporting Items for Systematic Reviews and Meta-Analyses (PRISMA) methodology and did not include protocol registration, a formal risk-of-bias assessment, or meta-analysis. This approach was considered appropriate because the available literature includes diverse study types, technical reports, regulatory documents, professional guidance, and early-stage clinical applications that are not readily comparable through quantitative synthesis. During revision, the reporting of this narrative review was refined using the Scale for the Assessment of Narrative Review Articles (SANRA) [16] as a quality-oriented framework, particularly with respect to the justification of the review, explicit aims, description of the literature search, appropriate referencing, scientific reasoning, and presentation of relevant evidence.

Data Sources and Search Strategy

A structured literature search was conducted in PubMed/MEDLINE, Scopus, Web of Science, and Google Scholar. Google Scholar was used to identify grey literature of scientific or professional relevance, including technical reports, guidelines, and regulatory documents. Additional searches were performed directly on the websites of professional societies and regulatory agencies relevant to nuclear medicine, radiopharmacy, and medical software governance, including the Society of Nuclear Medicine and Molecular Imaging (SNMMI), the European Association of Nuclear Medicine (EANM), the International Atomic Energy Agency (IAEA), the US Food and Drug Administration (FDA), and the European Medicines Agency (EMA).

Search terms were combined using Boolean operators, including AND and OR. The search strategy included the following terms: artificial intelligence, AI, machine learning, deep learning, neural network, nuclear medicine, PET, SPECT, quantification, theranostics, radiopharmacy, dosimetry, image reconstruction, denoising, segmentation, radioligand therapy, radiomics, regulation, ethics, and software as a medical device. Searches were conducted in English and Spanish. The review covered publications from January 2015 to October 2025, and the final bibliographic search was completed on November 20, 2025. Spanish-language searches were included to improve the retrieval of literature and policy documents relevant to Latin American and Costa Rican contexts, particularly for regulatory and implementation-related aspects. Because this study was designed as a critical narrative review rather than a systematic review, the search was documented at the level of databases consulted, time frame, languages, search concepts, eligibility criteria, and thematic domains rather than as a database-specific reproducible search log. Exact database-specific Boolean strings and record counts were not available in the original search documentation.

Eligibility Criteria

Sources were considered eligible if they were directly relevant to AI in nuclear medicine and contributed to the clinical, technical, radiopharmacy, regulatory, ethical, or pharmacotherapeutic focus of the review. Eligible sources included original studies, whether retrospective, prospective, or multicenter; systematic or narrative reviews; technical studies evaluating reconstruction, denoising, quantification, segmentation, or dosimetry algorithms with demonstrated or plausible clinical implications; and guidelines, consensus statements, technical reports, or regulatory documents issued by recognized scientific societies or agencies. Publications written in English or Spanish were considered.

Sources were excluded if they were conference abstracts without available full text, duplicate records, publications without a clear relationship to nuclear medicine, strictly experimental studies without evident clinical transferability, records with insufficient information for critical interpretation, or studies focused exclusively on non-nuclear imaging modalities such as MRI or CT. Studies involving MRI or CT were considered only when these modalities were directly relevant to hybrid PET/CT, SPECT/CT, or PET/MRI workflows. 

Study Selection and Data Extraction

Source selection was conducted in sequential phases. First, titles and abstracts were screened to exclude clearly irrelevant publications. Second, full texts were assessed to confirm clinical, technical, regulatory, or radiopharmacy relevance. Third, sources were included according to the eligibility framework and the pharmacotherapeutic focus of the review. Uncertainties regarding eligibility were resolved through re-evaluation against the eligibility criteria, cross-referencing with related sources, and discussion among the author group until consensus was reached. Complementary website searches were used to identify potentially relevant professional, regulatory, and policy documents. Only documents that met the eligibility criteria and directly supported the synthesis were retained and cited.

For each included source, extracted information included study design, year of publication, study setting, primary objective, study population or dataset, imaging modality, AI architecture or technique, clinical or operational task, reported outcomes, author-reported limitations, potential conflicts of interest, regulatory status when available, and relevance to clinical practice, radiopharmacy, dosimetry, radiopharmaceutical quality, traceability, and patient safety. Source selection and data extraction were conducted by the author group. Disagreements or uncertainties regarding eligibility were resolved through re-evaluation against the inclusion criteria and discussion until consensus was reached.

Narrative Synthesis Approach

Given the heterogeneity of study designs, AI architectures, imaging modalities, validation strategies, and outcome metrics, the evidence was integrated through narrative synthesis rather than meta-analysis. Findings were organized into thematic domains reflecting the principal areas of AI development in nuclear medicine: historical evolution; image reconstruction, denoising, and quantitative stability; clinical applications in oncology, neurology, and cardiology; theranostics and personalized dosimetry; radiopharmacy operations; and ethical, regulatory, and governance considerations.

Within each domain, the synthesis prioritized recurrent findings, methodological inconsistencies, external validity concerns, and gaps relevant to future research and clinical implementation. The analysis also distinguished technical performance from demonstrated clinical benefit, with attention to dataset bias, retrospective design, limited sample size, lack of external validation, commercial dependence, and applicability to Latin American settings. Because this was a critical narrative review, no formal risk-of-bias assessment, PRISMA flow diagram, or meta-analysis was performed; these methodological constraints are addressed in the Limitations section.

Thematic synthesis of the evidence

Evolution of AI in Nuclear Medicine

The reviewed literature indicates a gradual shift in AI applications in nuclear medicine from technically oriented image-processing tasks toward broader clinical, theranostic, radiopharmacy-related, and governance-focused uses [5-10,15,17-21]. Early work primarily demonstrated the feasibility of deep learning for PET and SPECT reconstruction, denoising, and post-reconstruction image enhancement. These studies suggested that reduced-count or shorter-acquisition protocols could be technically feasible while maintaining diagnostic image quality and, in selected contexts, quantitative stability [11,12,23].

Subsequent research moved beyond proof-of-concept performance toward broader validation and early clinical integration. Multicenter studies evaluating low-count PET reconstruction across different scanner platforms suggested that deep learning-based reconstruction may retain performance outside isolated experimental settings [11,12]. In parallel, radiomics became increasingly integrated into PET and SPECT analysis, enabling the extraction of image-derived features potentially associated with tumor biology, treatment response, and cardiovascular risk [5,6].

From 2022 onward, the field expanded into more complex clinical and operational domains. AI-supported methods were increasingly described in theranostic workflows, where quantitative imaging and internal dosimetry are central to treatment planning [9,10]. In radiopharmacy, emerging applications included synthesis support, quality control, process monitoring, and inventory management [7,8]. At the same time, professional guidance and symposium reports placed greater emphasis on validation, transparency, governance, and responsible clinical deployment [15,24].

Image Reconstruction, Denoising, and Quantitative Stability

Image reconstruction and quantitative stabilization remain the most technically mature areas of AI application in nuclear medicine. Across the reviewed literature, several studies reported improvements in noise reduction, count recovery, and preservation of quantitative parameters in PET and SPECT studies acquired with reduced activity or shorter acquisition time [2,3,11,12,23]. Other studies reported that AI-assisted reconstruction produced images with quantitative characteristics comparable to those obtained under standard-time or full-count conditions, supporting potential use in technically constrained acquisition settings [10-12].

These improvements are clinically relevant because nuclear medicine depends on quantitative parameters such as standardized uptake value, lesion burden, volume-of-interest measurements, and radiomic features. However, algorithmic improvements in image appearance do not automatically imply stable clinical performance. Quantitative outputs may vary according to scanner type, acquisition settings, reconstruction parameters, and model training data [2,3,6,11,12,23]. Radiomics-based applications add further complexity because texture and heterogeneity features are sensitive to preprocessing and reconstruction conditions [5,6].

From a pharmacotherapeutic perspective, AI-assisted reconstruction may support shorter acquisitions or lower administered activity, but these possibilities require careful verification. Any algorithm that modifies quantitative parameters can influence longitudinal comparability, dosimetric interpretation, or radiopharmaceutical use. Therefore, implementation should include local validation, cross-platform evaluation, and multidisciplinary review of whether technical improvements preserve clinically meaningful quantitative integrity.

Clinical Applications by Domain

Clinical applications of AI in nuclear medicine are concentrated primarily in oncology, neurology, and cardiology. Across these domains, AI has been used to support lesion detection, automated segmentation, quantitative analysis, and reduction of interobserver variability, with PET and SPECT serving as the principal imaging modalities [1,5,13,14,25,26].

In oncology, CNN-based models have been applied to automated lesion detection, tumor segmentation, and tumor-burden quantification across different tracers and malignancies [13,14]. AI-assisted segmentation may improve consistency with expert delineation and reduce interobserver variability, which is relevant for staging, response assessment, and multidisciplinary treatment planning. Oncology, therefore, remains one of the most active areas of clinically oriented AI development in nuclear medicine [13,14].

In neurology, AI models applied to [18F]FDG-PET have been used to identify metabolic patterns associated with Alzheimer's disease and to assist differentiation among neurodegenerative conditions [25]. These approaches may support the recognition of subtle hypometabolic patterns that are difficult to assess consistently by visual interpretation alone. In cardiology, AI applications in myocardial perfusion imaging have focused on automated defect detection, attenuation correction, and integration of structural and functional information derived from SPECT/CT studies [26].

Although these applications are promising, the evidence remains constrained by dataset dependence, differences between PET and SPECT platforms, limited external validation, and scarce representation of Latin American settings [1,5,13,14,20,25,26]. Clinical adoption should therefore depend not only on diagnostic performance metrics but also on evidence that models improve reproducibility, patient management, and workflow safety within the institutional context in which they are deployed.

Theranostics and Personalized Dosimetry

Theranostics is among the most quantitatively demanding areas of contemporary nuclear medicine. AI has been proposed as a tool to support quantitative imaging workflows, time-activity curve modeling, absorbed-dose estimation, and treatment planning in radioligand therapy [9,10]. These applications are particularly relevant because theranostics integrates diagnostic imaging, radiopharmaceutical selection, individualized activity planning, and therapeutic monitoring within a single molecular framework.

The reviewed literature highlights the technical promise of AI-supported dosimetry while also underscoring the need for stronger prospective validation [9,10]. Current evidence remains heterogeneous, with variable dosimetric methods, limited patient numbers, and insufficient direct linkage between AI-derived dose estimates and clinically meaningful outcomes such as tumor response or treatment-limiting toxicity. This gap is especially important because systematic error in dosimetric estimation could influence activity selection and patient safety.

For radiopharmacists and hospital pharmacists, theranostic workflows emphasize the need to preserve the quantitative chain from diagnostic acquisition to therapeutic planning. AI-based modifications to acquisition, reconstruction, segmentation, or post-processing may influence absorbed-dose estimation and pharmacotherapeutic decision-making. Accordingly, AI-derived dosimetry should be implemented only within transparent validation frameworks, institutionally defined protocols, and multidisciplinary risk-benefit assessment that includes pharmacist participation.

AI in Radiopharmacy Operations

Radiopharmacy has emerged as an operational domain for AI applications in nuclear medicine [7,8]. The reviewed literature identifies potential uses in radiochemical synthesis optimization, quality control support, process monitoring, and inventory or demand forecasting. These applications may be particularly valuable in workflows involving short-lived radionuclides, narrow production windows, and strict quality assurance requirements.

However, evidence in this area remains fragmented and less mature than the evidence supporting image reconstruction or selected clinical imaging tasks [7,8]. Many proposed applications have undergone limited validation under routine hospital conditions, where workload variability, equipment differences, staff training, and supply constraints may influence performance. Evidence is also limited regarding the integration of AI tools with institutional traceability systems, radiopharmaceutical quality management, and local regulatory workflows.

**Figure 1:**
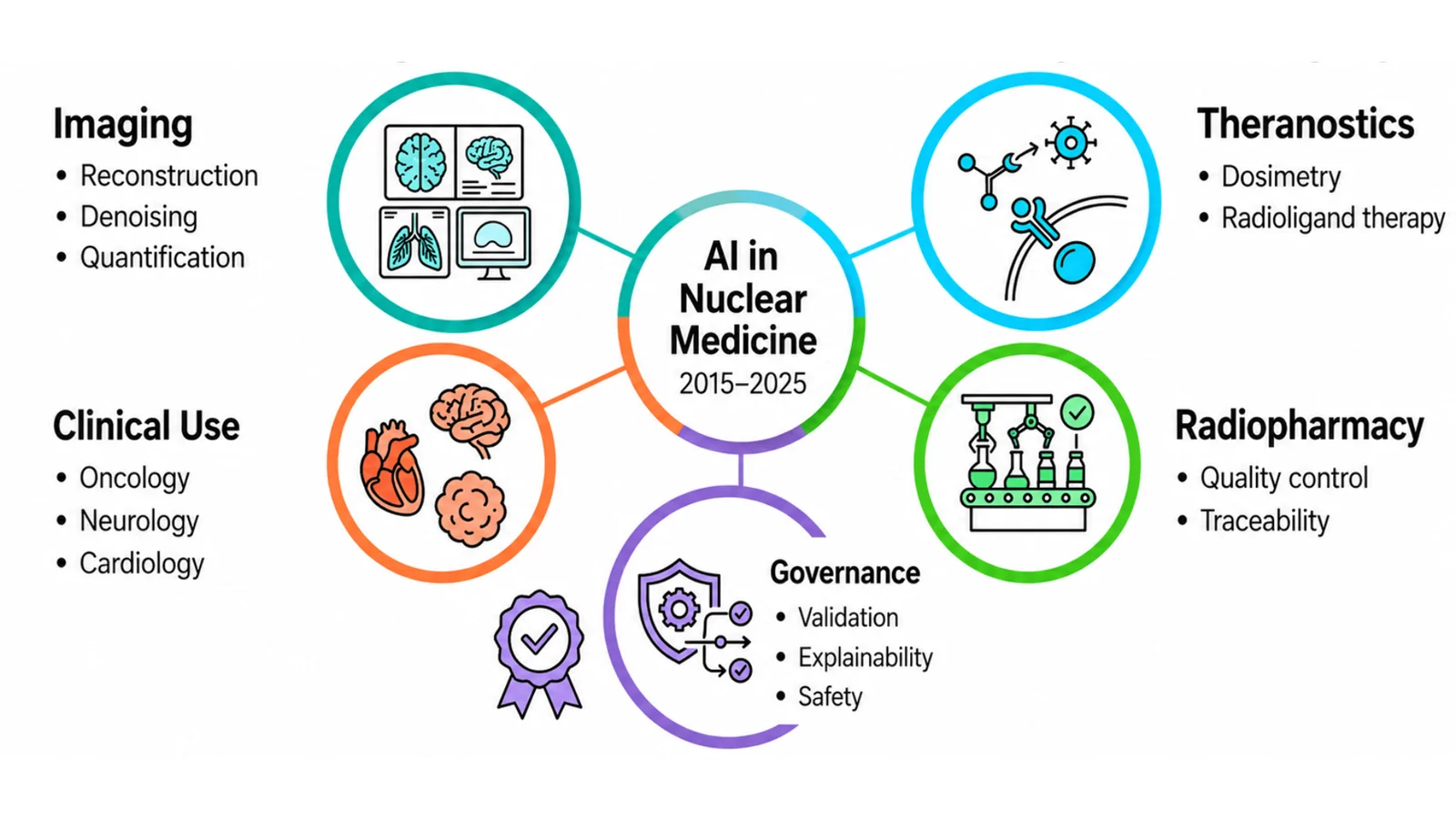
Key review highlights on AI in nuclear medicine AI: artificial intelligence This visual summary presents the main domains identified in the narrative review: imaging applications, clinical use, theranostics, radiopharmacy, and governance. Across these areas, the reviewed literature describes an expanding role for AI in reconstruction, denoising, quantification, segmentation, dosimetry, and workflow optimization while emphasizing the need for validation, explainability, patient safety, and multidisciplinary oversight. The figure was created manually by the authors using Canva (Canva Pty Ltd., Sydney, Australia). The figure does not contain generative AI-generated content.

The pharmacotherapeutic relevance of this domain is substantial. AI-supported process optimization may reduce technical errors and improve operational efficiency, but it may also introduce new risks if algorithmic outputs are adopted without independent verification. Radiopharmacist oversight is therefore essential to preserve radiopharmaceutical quality, sterility, activity accuracy, traceability, and compliance with local regulatory requirements. The main domains identified in this thematic synthesis, including imaging applications, clinical use, theranostics, radiopharmacy, and governance, are summarized in Figure 1.

Ethical, Regulatory, and Governance Landscape

Ethical, regulatory, and governance considerations have become increasingly prominent as AI tools move from experimental development toward clinical and operational implementation in nuclear medicine [15,17,18,22]. International guidance and professional discussions, including the RELAINCE guidelines and reports from AI-focused nuclear medicine symposia, emphasize the need for rigorous validation, transparency, task-specific performance assessment, independent evaluation, and post-implementation monitoring [15,24].

Several ethical issues are particularly relevant to nuclear medicine. First, many deep learning systems remain difficult to interpret, raising questions of accountability when algorithmic outputs influence quantitative imaging, dosimetry, or clinical decision-making [15,17,18]. Second, model performance may be affected by dataset representativeness, scanner variability, acquisition protocols, and population-level differences, creating the possibility of unequal performance across institutions and health systems [19,21]. Third, commercially developed tools require independent evaluation to determine whether reported performance is reproducible under local clinical conditions [8,15,20].

Regulatory development remains uneven across jurisdictions. Although international frameworks for evaluating AI in medical imaging continue to evolve, Latin American settings, including Costa Rica, still have limited AI-specific healthcare frameworks and may face additional barriers related to infrastructure, local validation capacity, equipment heterogeneity, and workforce training [15,17-22]. In these contexts, institutional governance, multidisciplinary oversight, and local validation should be regarded as practical requirements for safe adoption rather than optional quality measures. Table 1 summarizes the principal thematic findings, recurring limitations, research priorities, and pharmacotherapeutic implications identified across the reviewed literature.

**Table 1:** Summary of thematic findings, limitations, research priorities, and pharmacotherapeutic implications identified in the reviewed literature CNN: convolutional neural network; GAN: generative adversarial network; AI: artificial intelligence; PSMA: prostate-specific membrane antigen; PET: positron emission tomography; SPECT: single-photon emission computed tomography

Domain	Principal findings	Recurring limitations	Research priorities and pharmacotherapeutic implications
Image reconstruction and quantitative stability	Increasing use of CNN, U-Net, and GAN architectures; improved denoising, count recovery, low-count reconstruction, and preservation of quantitative parameters [2,3,6,11,12,24]	Scanner and protocol variability; radiomic instability; limited external validation; insufficient assessment of clinical impact	Multicenter harmonization; robust models across scanners and protocols; explainable AI; pharmacist-supported verification of quantitative stability and dosimetric implications
Clinical applications	Automated segmentation, lesion detection, PSMA PET quantification, Alzheimer disease pattern recognition, and cardiac SPECT support [1,5,13,14,25,26]	Dataset dependence; PET/SPECT differences; limited validation in diverse populations; scarce Latin American evidence	Prospective validation; multimodal integration; assessment in heterogeneous health systems; evaluation of impact on therapeutic decisions and radiopharmaceutical selection
Theranostics and personalized dosimetry	Emerging use of AI for quantitative imaging, time-activity modeling, absorbed-dose estimation, and radioligand therapy planning [9,10]	Small datasets; heterogeneous dosimetry methods; limited prospective evidence; weak linkage with outcomes such as response or toxicity	Standardized dosimetric benchmarks; radiopharmaceutical-specific models; explainability; pharmacist participation in dose verification and risk-benefit assessment
Radiopharmacy operations	Potential applications in synthesis support, quality control, process monitoring, and inventory management [7,8]	Fragmented evidence; limited validation in routine hospital conditions; uncertain integration with traceability systems	Real-world validation; predictive quality assurance; demand forecasting; pharmacist-led governance of radiopharmaceutical quality and traceability
Ethical, regulatory, and governance landscape	Growing emphasis on transparency, task-specific validation, independent evaluation, RELAINCE guidance, and post-implementation monitoring [15,17,18,22,24]	Opaque models; uneven regulation; limited Latin American frameworks; uncertain accountability; scarce algorithmic auditing	Local validation frameworks; continuous auditing; multidisciplinary governance; regionally applicable standards for safe AI implementation

Discussion

Integration of Main Findings

This review shows that AI in nuclear medicine has evolved from a predominantly technical field into a broader set of applications involving image reconstruction, clinical decision support, personalized dosimetry, radiopharmacy operations, and governance [6-8,11-13,23]. The most frequently reported evidence remains concentrated in image reconstruction and quantitative stabilization, where several studies reported improvements in denoising, count recovery, and preservation of clinically relevant quantitative parameters [2,3,10-12,23].

Clinical applications in oncology, neurology, and cardiology have also advanced, particularly in automated segmentation, pattern recognition, and standardization of image interpretation [13,14,25,26]. By contrast, theranostics, personalized dosimetry, and radiopharmacy operations are supported by a more fragmented and methodologically uneven evidence base [7-10]. This imbalance suggests that technical feasibility has progressed faster than clinically generalizable implementation.

The central implication is that AI tools in nuclear medicine should be evaluated according to their intended use. A model that improves image appearance, shortens acquisition time, or automates segmentation may still require separate validation for quantitative stability, therapeutic relevance, and local workflow safety. This distinction is essential in nuclear medicine, where quantitative outputs can influence administered activity, dosimetry, longitudinal assessment, and therapeutic decision-making.

Technical Performance Versus Clinical Generalizability

A recurrent finding across domains is the gap between favorable technical performance and robust external validity. Many studies report favorable algorithmic performance under controlled conditions, but such results do not necessarily translate into stable performance across institutions, scanner platforms, acquisition protocols, tracers, or patient populations [2,6,11-14,20,23,25,26]. This concern is particularly important in nuclear medicine because small technical variations can affect quantitative parameters with clinical and pharmacotherapeutic implications.

In reconstruction studies, AI-assisted methods may support lower-count acquisitions, but performance remains dependent on scanner hardware, reconstruction settings, and protocol standardization [6,11,12]. In clinical applications, model transferability may be constrained by imaging modality, radiotracer, disease prevalence, and dataset composition [1,5,13,14,25,26]. In theranostics, variation in dosimetric methodology further limits comparability across studies and complicates the definition of implementation-ready standards [9,10].

Accordingly, technical performance should be interpreted as one component of readiness, not as sufficient justification for clinical deployment. Before implementation, AI models should demonstrate reproducibility, clinically meaningful benefit, compatibility with existing quality assurance processes, and acceptability within local regulatory and operational conditions.

Pharmacotherapeutic Implications and the Role of the Radiopharmacist

The pharmacotherapeutic relevance of AI in nuclear medicine is most apparent in workflows where algorithmic outputs may influence administered activity, quantitative follow-up, radiopharmaceutical preparation, or treatment planning. AI-assisted reconstruction may support lower-dose or shorter-acquisition protocols, but these benefits depend on the preservation of quantitative comparability and verification that denoising does not distort clinically relevant parameters [2,3,10-12,24].

In theranostics, AI-derived dosimetry may contribute to radioligand therapy planning, but the current evidence remains insufficient to support broad unsupervised adoption [9,10]. In radiopharmacy operations, AI may improve synthesis support, quality control, and inventory planning; however, limited validation under routine hospital conditions requires careful institutional assessment before implementation [7,8].

Radiopharmacists are therefore positioned as key contributors to the governance of AI-enhanced nuclear medicine workflows. Their role extends beyond radiopharmaceutical preparation to include assessment of quantitative stability, verification of AI-supported quality processes, interpretation of dosimetric implications, and participation in multidisciplinary risk assessment. In workflows where image-derived parameters are linked to radiopharmaceutical administration and patient safety, radiopharmacist oversight is an essential component of responsible clinical governance [7-10,15,17,18].

Implications for Latin America

The regional implications of AI adoption are substantial. Published discussions of AI implementation in medical imaging indicate that infrastructure, workflow, training, and validation conditions in low- and middle-income settings may differ considerably from those of high-volume centers in North America or Europe [21]. In Latin America, heterogeneity in equipment, institutional capacity, regulatory maturity, and access to specialized personnel may limit the transferability of AI tools trained or validated elsewhere [15,17-22].

For Costa Rica and comparable settings, the limited development of AI-specific healthcare frameworks reinforces the need for institution-level validation and governance [21,22]. Adoption should account for local scanner platforms, acquisition protocols, radiopharmaceutical supply chains, workforce training, and data representativeness. These considerations are especially important in nuclear medicine because quantitative inconsistency can affect not only image interpretation but also dosimetry, radiopharmaceutical use, and longitudinal therapeutic decisions.

At the same time, regional implementation offers an opportunity to generate context-specific evidence. Local validation networks, multicenter collaborations, and pharmacist-led quality initiatives could help produce data that better reflect Latin American populations and health system constraints. Such efforts would reduce dependence on extrapolated performance claims and support safer, more context-appropriate AI adoption.

Future Research Priorities

Future research should address the persistent gap between technical optimization and clinical utility. In image reconstruction, multicenter harmonization studies are needed to determine whether AI models maintain quantitative stability across scanners, institutions, tracers, and acquisition protocols [3,6,11]. In clinical applications, prospective studies should evaluate whether AI improves diagnostic decision-making, reproducibility, therapeutic planning, and patient-relevant outcomes in diverse populations [1,5,13,14,20,25,26].

In theranostics, future work should prioritize explainable models, standardized dosimetric benchmarks, radiopharmaceutical-specific validation, and prospective evaluation of AI-assisted dosimetry in relation to response and toxicity [9,10]. In radiopharmacy, studies should test AI-supported synthesis, predictive quality control, and inventory planning under routine operational variability rather than only under controlled conditions [7,8]. Across all domains, research should incorporate transparent reporting, independent validation, conflict-of-interest assessment, and evaluation in settings beyond highly resourced centers [8,15,17-20,22].

Limitations

This review has limitations inherent to its critical narrative design. Although the literature search was structured and the manuscript was refined using SANRA as a quality-oriented framework for narrative reviews, the study was not designed as a systematic review. Therefore, it did not include a formal risk-of-bias assessment, PRISMA flow diagram, meta-analysis, or pooled quantitative synthesis. Consequently, the findings should be interpreted as an integrative and critical synthesis of the available evidence rather than as a formal quantitative quality rating of individual studies. An additional limitation is that exact database-specific Boolean strings, record counts by source, duplicate-removal counts, and record-level exclusion reasons were not preserved in the original search documentation. Therefore, although the review process was structured and the search concepts are reported, the source identification process cannot be replicated with the same level of precision expected from a systematic review. This limitation is consistent with the critical narrative design of the article and should be considered when interpreting the scope of the synthesis.

The review period was restricted to publications from 2015 to 2025, which supports a focus on contemporary AI applications in nuclear medicine but may have excluded earlier foundational studies or publications appearing after the search closure date. In addition, the included literature was heterogeneous in study design, imaging modality, AI architecture, validation strategy, and reported outcomes. Many studies were retrospective, single-center, technically focused, or validated within limited datasets, which restricts generalizability.

Some thematic domains, particularly theranostic dosimetry, radiopharmacy operations, and Latin American implementation, were supported by a smaller number of directly relevant sources. These areas were therefore interpreted as emerging domains rather than mature bodies of evidence. The pharmacotherapeutic analysis was based on published literature, professional guidance, and regulatory documents rather than primary field data. Accordingly, local institutional conditions, regulatory environments, infrastructure, and workforce capacity may influence the applicability of the conclusions.

## Conclusions

Over the past decade, AI has become increasingly relevant to nuclear medicine, with applications spanning image reconstruction, denoising, quantitative stabilization, automated clinical tasks, personalized dosimetry, and radiopharmacy operations. These developments may improve diagnostic precision, workflow efficiency, radiopharmaceutical management, and individualized radioligand therapy, provided that technical gains translate into reproducible clinical value.

The path from algorithmic performance to responsible clinical implementation requires more than favorable technical metrics. AI tools in nuclear medicine should undergo robust multicenter validation, transparent task-specific evaluation, assessment of local applicability, adaptive regulatory oversight, and continuous multidisciplinary governance. This is particularly important because AI-generated outputs may influence quantitative imaging, administered activity, dosimetry, longitudinal assessment, and therapeutic decision-making.

For Costa Rica and Latin America, where regulatory frameworks, infrastructure, and implementation capacity remain uneven, safe adoption will depend on local validation, institutional governance, workforce training, and context-specific evidence generation. Radiopharmacists are strategically positioned to contribute to this process by overseeing radiopharmaceutical quality, traceability, dosimetric implications, and pharmacotherapeutic safety. Ultimately, the value of AI in nuclear medicine will depend not on uncritical adoption but on evidence-based, transparent, and patient-centered implementation.
